# Machine learning-based prediction model for cognitive impairment risk in patients with chronic kidney disease

**DOI:** 10.1371/journal.pone.0324632

**Published:** 2025-06-05

**Authors:** Meng Cao, Bixia Tang, Liwei Yang, Jing Zeng

**Affiliations:** 1 School of Nursing, Chengdu Medical College, Chengdu, China; 2 School of Nursing, Sichuan Vocational College of Health and Rehabilitation, Zigong, China; 3 Department of Urology, Chengdu Seventh People’s Hospital, Chengdu, China; University of Luzon, PHILIPPINES

## Abstract

**Background:**

The high prevalence of cognitive impairment (CI) in Chronic kidney disease (CKD) patients impacts their quality of life and prognosis, yet risk prediction models for CI in this population remain underexplored.

**Objective:**

This study aimed to develop a risk prediction model for CI in CKD patients using machine learning algorithms, with the objective of enhancing risk prediction accuracy and facilitating early intervention.

**Methods:**

A total of 415 CKD patients from the 2015 China Health and Retirement Longitudinal Survey (CHARLS) dataset were included in this study. Participants were categorized into two groups: the CI group (n = 53) and the non-CI group (n = 362). Binary logistic regression, encompassing both univariate and multivariate analyses, was conducted to identify influencing factors. Subsequently, a CI risk prediction model was constructed using four machine learning algorithms: Support Vector Machine (SVM), Random Forest (RF), Neural Network (NN), and Logistic Regression (LR). The optimal model was further assessed for predictor importance utilizing the SHAP method and deployed on a web platform using the Streamlit library.

**Results:**

Logistic regression analysis identified age, hemoglobin concentration, education level, and social participation as significant factors influencing CI. Models based on NNET, RF, LR, and SVM algorithms were developed, achieving AUC of 0.918, 0.889, 0.872, and 0.760, respectively, on the test set. Calibration curves demonstrated that all models were well-calibrated. Among these, the NNET model exhibited the highest predictive performance. According to the SHAP analysis of the optimal model, the most influential predictors are age, education level, and hemoglobin concentration.

**Conclusion:**

Machine learning models are valuable tools for predicting the risk of CI in CKD patients and can assist healthcare professionals in developing appropriate intervention strategies.

## 1. Introduction

Chronic kidney disease (CKD) has emerged as a significant global public health challenge. Epidemiological studies indicate that the worldwide prevalence of CKD is approximately 14.3%, with a prevalence of 10.8% in China [1–4]. Moreover, CKD is projected to become the fifth leading cause of mortality globally by 2040 [5].

Cognition is the core of human mental activity, involving the processes of knowledge acquisition and understanding [6]. Cognitive impairment (CI) is a neurodegenerative aging process, characterized by impairments in language, attention, memory, reasoning, judgment, and visual perceptual functions [7].

It has been estimated that CI affects approximately 10% to 40% of CKD patients [8]. CI reduces patients’ quality of life, impacts their adherence to treatment, and increases the risk of death [9]. Therefore, predicting the risk of CI in the CKD population is crucial. However, there are relatively few studies on CI risk prediction models for CKD patients both domestically and internationally, with most focusing on exploring

CI influencing factors. Most existing models are designed for maintenance hemodialysis patients and predominantly use traditional logistic regression algorithms. This study will apply four machine learning algorithms (NNET, RF, LR, and SVM) to construct a CI risk prediction model for the CKD population using data from the China Health and Retirement Longitudinal Study (CHARLS) database. The model with the best predictive performance will be identified through comparison, its predictors’ importance will be evaluated using the SHAP method, and the optimal model will be deployed on a web page using the Streamlit library. This approach aims to improve CI risk prediction in the CKD population and provide a basis for early intervention.

## 2. Methods

### 2.1. Data source

The data for this study were sourced from the China Health and Retirement Longitudinal Study (CHARLS). CHARLS is led by the National Development Research Institute of Peking University. It aims to investigate health and aging issues in the middle-aged and elderly population in China. A nationwide baseline survey was conducted from 2011 to 2012, employing random sampling across 150 county-level units, surveying 10,257 households with at least one resident aged 45 or older, totaling 17,708 individuals. Follow-up surveys were conducted in 2013 and 2015, encompassing 20,284 individuals [10]. The database includes comprehensive health and socioeconomic information, such as renal disease history and cognitive functioning test results, among other variables.The Ethics Committee of Peking University reviewed and approved CHARLS (Ethics No. IRB00001052–11015). All participants or their proxies provided signed informed consent.The data analyzed in this study were obtained from CHARLS and did not require additional ethical review by the investigator’s affiliated institution. Secondary analyses did not necessitate additional institutional review board approval.

### 2.2. Participants

**Fig 1:**
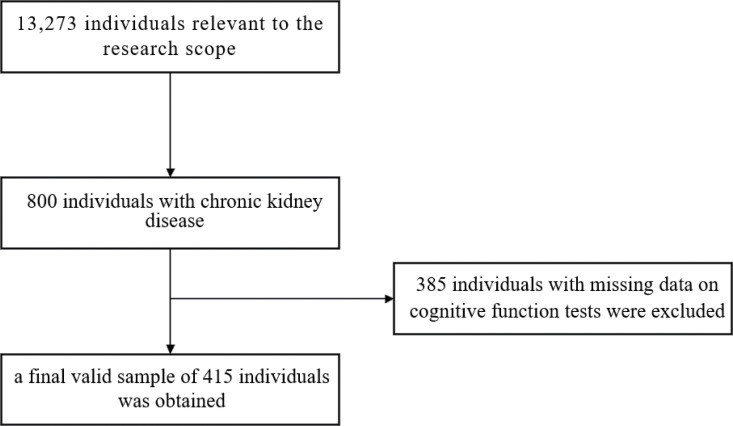
Sample screening flowchart.

Data were obtained from the 2015 CHARLS database. We accessed the database on April 10, 2024, and included 13,273 individuals relevant to the scope of the study, screened 800 individuals with chronic kidney disease, and excluded 385 individuals with missing information on cognitive function tests, resulting in a final valid sample of 415 individuals (Fig 1).

### 2.3. Relevant definition

Chronic Kidney Disease (CKD) is assessed based on the estimated glomerular filtration rate (eGFR), with CKD defined as eGFR < 60 mL/(min·1.73 m²) according to the Expert Consensus on Renal Disease [11]. eGFR is calculated using the following MDRD equation [12].

Male:eGFR (MDRD) [mL/(min·1.73 m^2^)] = 175*Scr(mg/dL)^1.154*Age^0.203

Female:eGFR (MDRD) [mL/(min·1.73 m^2^)] = 175*Scr(mg/dL)^1.154*Age^0.203*0.742

### 2.4. Outcome variable

CI determination: The 2015 CHARLS study did not provide a definitive diagnosis of cognitive impairment. Referring to previous literature [13], cognitive function was assessed using two indicators: mental status and situational memory capacity, derived from the Health Status and Functioning Questionnaire. The mental status test evaluated the perception of the day of the year, day of the week, and season, along with numeracy and graphing abilities. Each correct response was awarded 1 point, with a total score ranging from 0 to 11. The situational memory test involved word recall, where participants were tasked with recalling two sets of 10 words at two different times. Each correctly recalled word scored 1 point, and the average score of the two recalls represented the final situational memory ability, with a total score range of 0–10. The cognitive functioning score was the sum of the mental status and situational memory scores, with a total possible range of 0–21. CI was defined as the lowest 10% of the population based on this total score.

### 2.5. Predictor variables

A total of 21 potential factors were extracted by combining all relevant factors reported in recent studies and considering the accessibility of variables in the database: (1) demographic characteristics—gender, age, education level, marital status [14]; (2) health behavior factors—smoking, alcohol consumption [7], self-assessment of health, sleep duration, depression status [15], and social participation [16]; (3) physical examination indicators—BMI [7]; (4) laboratory test indicators—blood creatinine, hemoglobin, glycosylated hemoglobin, total cholesterol, triglycerides, high-density lipoprotein cholesterol, low-density lipoprotein cholesterol, fasting blood glucose [8], ultrasensitive C-reactive protein [15], and glomerular filtration rate. Due to the proportion of missing data for smoking and self-rated health exceeding 20% in the database [17], these two variables were excluded. The remaining 19 variables were selected for inclusion in the study. After conducting univariate and multivariate binary logistic regression analysis, variables with p < 0.2 were included in the model construction.

Depression status was assessed using 10 questions: (1) eight negative mood items evaluating whether the individual was bothered by small things, had difficulty concentrating, felt depressed, struggled to accomplish tasks, experienced fear, had trouble sleeping, felt lonely, or was unable to get along with others; and (2) two positive mood items assessing whether the individual felt hopeful about the future and experienced happiness. Each question was scored on a 4-point scale (0 = rarely or not at all, 1 = not very much, 2 = sometimes or half the time, 3 = most of the time), with reverse scoring applied to the two positive mood items. The total scores from all 10 questions were summed to create a composite index of depressive symptoms, ranging from 0 to 30 points. Higher scores indicate greater levels of depression and lower mental health status among older adults.

Social participation data were derived from 11 activities listed in the CHARLS questionnaire, which included items such as “Have you engaged in any of the following activities in the past month: interacting with friends, playing mahjong, or playing cards,” among others. Each activity was scored based on the frequency of participation: a value of 3 was assigned if the respondent participated almost daily, 2 if they participated almost weekly, and 1 if participation was infrequent during the month. The scores for all 11 activities were summed to calculate the total social participation score, with higher scores indicating greater levels of social participation [18].

Body mass index (BMI) was calculated using the formula:BMI = weight (kg)/ height (m)^2 [19].

### 2.6. Statistical analysis

Continuous variables were expressed as mean ± standard deviation, and comparisons between groups were conducted using independent samples t-tests. Categorical variables were presented as n (%), with group comparisons performed using chi-square tests. Missing values were imputed using the MICE package in R Studio 4.3.1. Binary logistic regression, including univariate and multivariate analyses, was applied to screen for CI influencing factors in CKD patients, with variables exhibiting P < 0.2 included in the subsequent model construction.

The study sample consists of 415 participants, with a relatively small data volume. The predictor variables include both multicategorical and continuous variables, while the outcome variable is dichotomous. RF is a decision tree-based model, SVM is based on support vector machines, LR is a linear model, and NNET is a neural network model. Given the limited data in this study, complex models like deep learning are not suitable. Therefore, four machine learning algorithms—NNET, RF, LR, and SVM—were selected for model construction.These algorithms are well-suited for handling diverse data types, as they can capture complex nonlinear relationships and demonstrate stable performance across varying data contexts.

The dataset was split into training and test sets using the `train_test_split` function (75–25 ratio) in Python 3.10, and the predictor variables were one-hot encoded and normalized. Given the data imbalance (with a Positive: Negative sample ratio of approximately 7:1), the training dataset was oversampled using the SMOTE (Synthetic Minority Oversampling Technique) method. Specifically, minority class samples are randomly duplicated to balance the class distribution, enhancing the model’s ability to identify the minority class. Meanwhile, to maintain fairness, this adjustment is not applied to the test set.The model was trained and parameters optimized via 5-fold cross-validation and grid search. Model performance was evaluated based on AUC, accuracy, recall, specificity, precision, and F1 score. Model calibration was assessed using calibration curves. The model with the highest AUC was selected as the best-performing model. The importance of its predictors was evaluated using the SHAP method, and the optimal model was deployed on the web page using the Streamlit library.

## 3. Results

### 3.1. Description of the characteristics of the research sample

The 415 CKD participants included 53 patients with CI,362 patients with normal cognition, and Table 1 describes the differences in characteristics between the two. Compared with cognitively normal CKD patients, CKD patients with CI were older (mean age 71.7 + 8.0 years), had lower hemoglobin, and poorer social participation. The proportion of CKD patients with CI was higher in those who were illiterate and had not completed elementary school than in those who were cognitively normal; the proportion of CKD patients with CI was lower in those who had completed elementary school and junior high school than in those who were cognitively normal.

**Table 1:** Descriptive statistics for variables.

variable	Description	no (N = 362)	yes (N = 53)	OR (univariable)	OR (multivariable)
Gender	male	213 (58.8%)	23 (43.4%)		
	female	149 (41.2%)	30 (56.6%)	1.86 (1.04–3.34, p = .036)	0.88 (0.37–2.11, p = .777)
Age	Mean ± SD	64.8 ± 9.9	71.7 ± 8.0	1.08 (1.05–1.12, p < .001)	1.07 (1.02–1.13, p = .009)
Marital status	Married	296 (81.8%)	37 (69.8%)		
	Married but Separated	13 (3.6%)	0 (0%)	0.00 (0.00-Inf, p = .989)	0.00 (0.00-Inf, p = .995)
	Divorced	5 (1.4%)	0 (0%)	0.00 (0.00-Inf, p = .993)	0.00 (0.00-Inf, p = .997)
	Widowed	45 (12.4%)	16 (30.2%)	2.84 (1.46–5.53, p = .002)	1.08 (0.45–2.61, p = .868)
	unmarried	3 (0.8%)	0 (0%)	0.00 (0.00-Inf, p = .995)	0.00 (0.00-Inf, p = .998)
Drink	frequent	102 (28.2%)	11 (20.8%)		
	Infrequent	29 (8%)	4 (7.5%)	1.28 (0.38–4.32, p = .692)	
	Non-drinker	231 (63.8%)	38 (71.7%)	1.53 (0.75–3.10, p = .244)	
bl_crea	Mean ± SD	1.5 ± 0.4	1.4 ± 0.4	0.61 (0.29–1.30, p = .200)	0.71 (0.28–1.76, p = .455)
bl_hgb	Mean ± SD	13.5 ± 1.8	12.4 ± 1.6	0.71 (0.59–0.84, p < .001)	0.75 (0.60–0.93, p = .010)
bl_hbalc	Mean ± SD	5.9 ± 0.6	6.0 ± 0.6	1.26 (0.79–2.01, p = .333)	
bl_cho	Mean ± SD	184.4 ± 39.1	180.0 ± 40.3	1.00 (0.99–1.00, p = .443)	
bl_tg	Mean ± SD	142.1 ± 70.2	153.0 ± 82.7	1.00 (1.00–1.01, p = .304)	
bl_hdl	Mean ± SD	48.9 ± 10.7	47.1 ± 10.3	0.98 (0.96–1.01, p = .246)	
bl_ldl	Mean ± SD	104.2 ± 30.1	99.0 ± 29.1	0.99 (0.98–1.00, p = .235)	
bl_glu	Mean ± SD	101.7 ± 17.9	103.2 ± 17.5	1.00 (0.99–1.02, p = .590)	
bl_crp	Mean ± SD	2.6 ± 2.3	2.9 ± 2.5	1.05 (0.93–1.18, p = .440)	
eGFR	Mean ± SD	45.7 ± 12.3	45.4 ± 11.9	1.00 (0.97–1.02, p = .865)	
Educational level	Illiterate	40 (11%)	26 (49.1%)		
	Did not finish primary school	69 (19.1%)	17 (32.1%)	0.38 (0.18–0.78, p = .009)	0.34 (0.15–0.76, p = .008)
	Home school	6 (1.7%)	1 (1.9%)	0.26 (0.03–2.25, p = .220)	0.15 (0.01–1.69, p = .125)
	Elementary school	106 (29.3%)	8 (15.1%)	0.12 (0.05–0.28, p < .001)	0.11 (0.04–0.30, p < .001)
	Middle school	100 (27.6%)	1 (1.9%)	0.02 (0.00–0.12, p < .001)	0.03 (0.00–0.20, p < .001)
	High school	22 (6.1%)	0 (0%)	0.00 (0.00-Inf, p = .990)	0.00 (0.00-Inf, p = .993)
	Vocational school	11 (3%)	0 (0%)	0.00 (0.00-Inf, p = .993)	0.00 (0.00-Inf, p = .995)
	Associate degree	4 (1.1%)	0 (0%)	0.00 (0.00-Inf, p = .996)	0.00 (0.00-Inf, p = .997)
	Bachelor’s degree	4 (1.1%)	0 (0%)	0.00 (0.00-Inf, p = .996)	0.00 (0.00-Inf, p = .997)
BMI	Mean ± SD	18.9 ± 1.2	19.7 ± 1.2	1.63 (1.29–2.08, p < .001)	1.14 (0.84–1.55, p = .405)
Social participation	Mean ± SD	2.5 ± 2.7	1.4 ± 1.7	0.82 (0.70–0.95, p = .007)	0.85 (0.71–1.01, p = .072)
Depression	Mean ± SD	7.6 ± 5.9	8.3 ± 4.5	1.02 (0.97–1.07, p = .384)	
Sleep Time	Mean ± SD	6.3 ± 1.8	6.1 ± 2.2	0.94 (0.81–1.10, p = .459)	

Mean ± SD, mean ± standard deviation; bl_crea, blood creatinine; bl_hgb, hemoglobin; bl_hbalc, glycosylated hemoglobin; bl_cho, total cholesterol; bl_tg, triglycerides; bl_hdl, high-density lipoprotein cholesterol; bl_ldl, low-density lipoprotein cholesterol; bl_glu, fasting blood glucose; bl_crp, ultrasensitive C-reactive protein; eGFR, glomerular filtration rate.

### 3.2. The outcomes of univariate and multivariate analyses

After univariate and multivariate logistic regression analysis, no statistically significant differences were found between the two groups regarding gender, marital status, alcohol consumption, sleep duration, depression status, BMI, blood creatinine, glycosylated hemoglobin, total cholesterol, triglycerides, high-density lipoprotein cholesterol, low-density lipoprotein cholesterol, fasting blood glucose, ultrasensitive C-reactive protein, and glomerular filtration rate (P > 0.2). However, statistically significant differences (P < 0.2) were observed for age, education level, social participation, and hemoglobin (Table 1).

For further descriptive analysis, odds ratios (ORs) for each predictor variable were calculated using univariate and multivariate logistic regression (Table 1). Among the demographic characteristics, the analysis revealed that age was a risk factor for cognitive impairment (CI) in the CKD population (OR 1.07, 95% CI 1.02–1.13). Compared with illiterate CKD patients, those who had not completed elementary school, graduated from elementary school, and graduated from junior high school had a lower risk of developing CI (OR 0.34, 95% CI 0.15–0.76; OR 0.11, 95% CI 0.04–0.30; OR 0.03, 95% CI 0.00–0.20, respectively). Among the health behavior variables, CKD patients with good social engagement had a lower risk of CI compared to those with poor social engagement (OR 0.85, 95% CI 0.71–1.01). Among the laboratory indicators, CKD patients with higher hemoglobin levels had a lower risk of CI compared to those with lower hemoglobin levels (OR 0.75, 95% CI 0.60–0.93).

### 3.3. Comparison of predictive performance among machine learning algorithms.

**Fig 2:**
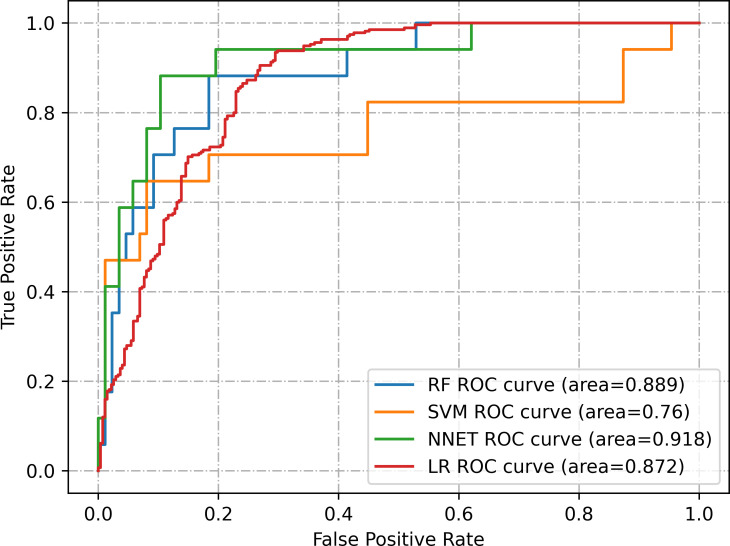
ROC curves for the test set of four models.

In this study, four machine learning models were developed to predict the probability of cognitive impairment (CI) in CKD patients. Fig 2 illustrates the predictive efficacy of the four models on the test set using ROC curves. Among these models, the NNET model (AUC = 0.918) demonstrated the highest effectiveness in predicting the occurrence of CI in CKD patients, followed by the RF model (AUC = 0.889), the LR model (AUC = 0.872), and the SVM model (AUC = 0.760).

Table 2 presents detailed performance metrics for the four models in both the training and test sets. In the test set, the RF model achieved the highest accuracy of 0.875, the NNET model exhibited the highest recall of 0.941, the SVM model attained the highest specificity of 0.931, and the LR model demonstrated the highest precision and F1 score of 0.75 and 0.834, respectively.

**Table 2:** Performance metrics of the models on the training and test set.

Models	AUC(95% CI)	Accuracy(95% CI)	Precision(95% CI)	Recall(95% CI)	Specificity(95% CI)	F1(95% CI)
LR Test	0.872 (0.8415, 0.9008)	0.813 (0.7781, 0.8454)	0.75 (0.7034, 0.7964)	0.938 (0.9073, 0.9652)	0.687 (0.6324, 0.7451)	0.834 (0.8012, 0.864)
LR Train	0.903 (0.7982, 0.9744)	0.75 (0.6634, 0.8269)	0.385 (0.2424, 0.5454)	0.882 (0.7058, 1.0)	0.724 (0.6304, 0.8148)	0.536 (0.375, 0.6865)
NNET Test	0.918 (0.8288, 0.977)	0.808 (0.7307, 0.8846)	0.457 (0.2972, 0.6285)	0.941 (0.8, 1.0)	0.782 (0.6962, 0.8681)	0.615 (0.439, 0.7636)
NNET Train	0.986 (0.9999, 1.0)	0.829 (0.8, 0.8581)	0.927 (0.8978, 0.9543)	0.775 (0.7303, 0.8206)	0.91 (0.8709, 0.9427)	0.844 (0.814, 0.873)
RF Test	0.889 (0.794, 0.9609)	0.875 (0.8076, 0.9326)	0.6 (0.3684, 0.8181)	0.706 (0.4736, 0.923)	0.908 (0.8481, 0.9651)	0.649 (0.4375, 0.8108)
RF Train	0.986 (0.9999, 1.0)	0.953 (0.9345, 0.969)	0.971 (0.9488, 0.9886)	0.937 (0.9072, 0.9644)	0.97 (0.9465, 0.9883)	0.954 (0.9347, 0.97)
SVM Test	0.76 (0.5952, 0.9112)	0.856 (0.7884, 0.9134)	0.571 (0.3076, 0.8461)	0.47 (0.25, 0.7142)	0.931 (0.8777, 0.9767)	0.516 (0.2962, 0.7058)
SVM Train	0.986 (0.9999, 1.0)	0.938 (0.9181, 0.9563)	0.982 (0.9659, 0.9962)	0.903 (0.8678, 0.9347)	0.98 (0.9628, 0.9959)	0.941 (0.9203, 0.9598)

**Fig 3:**
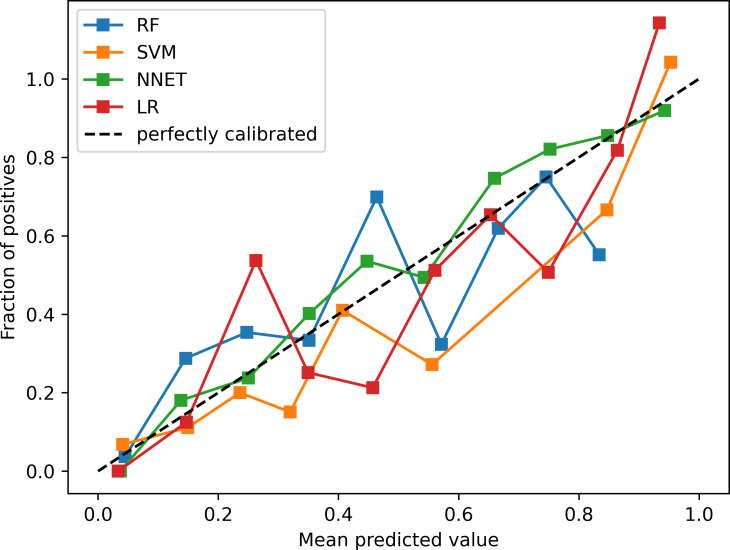
Calibration curves for the test set of four models.

The calibration curves of the four models are shown in Fig 3. The curves exhibit a high degree of overlap with the straight line of y = x, indicating good agreement between the predicted and actual occurrence risk values. Among the models, the NNET model demonstrates the best calibration.

### 3.4. Optimal model SHAP visualization interpretation.

**Fig 4:**
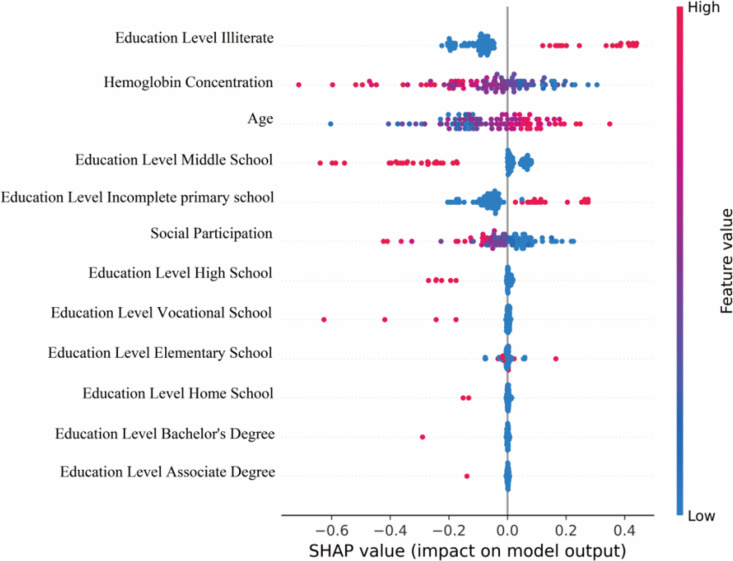
SHAP summary plot.

**Fig 5:**
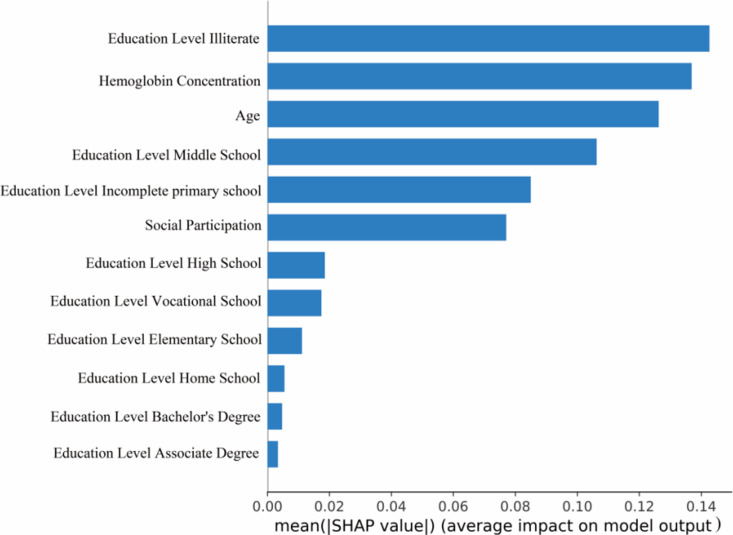
SHAP bar plot.

In the output model of this study, each predictor variable was translated into a contribution to the outcome for each patient. Specifically, the higher the SHAP value, the greater the risk of CI in CKD patients. The results indicated that the risk of CI increased progressively with the patient’s age, while a lower education level and reduced hemoglobin concentration were associated with a higher risk of CI (Fig 4). The significance of each variable in determining the outcome was quantified by the mean of the absolute SHAP values. The results indicated that age, education level, and hemoglobin concentration were the most influential predictors in the final model(Fig 5).

### 3.5. Deployment of the optimal model on the Web.

**Fig 6:**
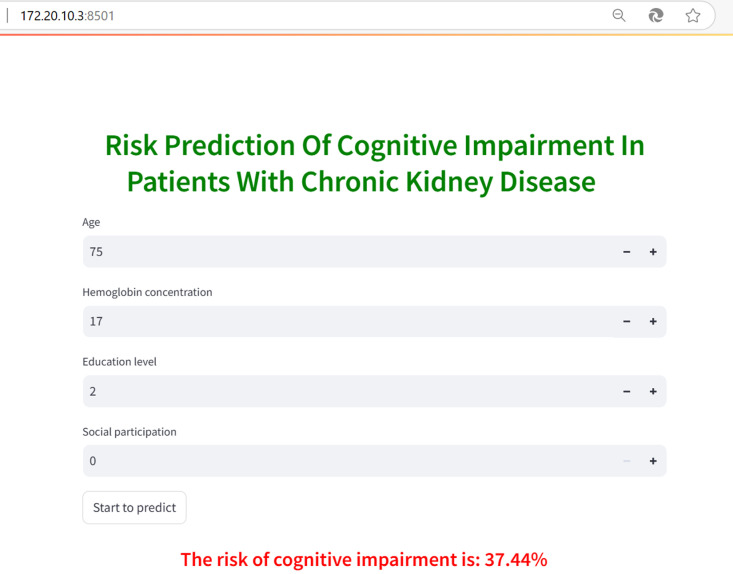
Display of the web prediction interface.

Using the Streamlit library, the optimal model is deployed on a web platform, with prediction results visually presented. Upon logging into the webpage through the local area network (LAN), users are prompted to enter the relevant index data and click the prediction button to initiate the process. The basic interface of the web application is shown in Fig 6.

## 4. Discussion

### 4.1. Analysis of variables associated with the risk of CI in patients with CKD.

#### 4.1.1. Age.

This study identifies age as a significant risk factor for CI in the CKD population. As age advances, CKD patients become increasingly vulnerable to cardiovascular risk factors, which can precipitate cerebrovascular pathology. Additionally, age-related physiological and pathological renal changes may elevate blood inflammatory marker levels, heightening susceptibility to cerebrovascular conditions [8] such as microembolism and hypoperfusion. These factors contribute to the development of lacunar cerebral infarcts and cerebral white matter degeneration, ultimately leading to CI. Therefore, healthcare professionals should prioritize early screening and rigorous monitoring of cognitive function in middle-aged and elderly CKD patients [20].

#### 4.1.2. Education level.

The study findings indicate that higher education levels are a protective factor for cognitive function in the CKD population. Individuals with greater educational attainment possess enhanced cognitive reserves and a more comprehensive array of cognitive skills and strategies, which enable them to better mitigate the impact of pathological changes associated with CKD. Moreover, higher education levels correlate with healthier behaviors [21], including balanced dietary patterns, regular physical activity, and reduced consumption of tobacco and alcohol, thereby lowering the risk of cardiovascular and other chronic diseases that can compromise cognitive function. Additionally, individuals with higher education levels often benefit from stronger social support networks and a more positive mental health status, which help alleviate the psychological stress linked to CKD, further safeguarding cognitive function. Hence, healthcare providers should consider patients’ educational backgrounds and tailor health education programs and interventions to address their specific needs effectively.

#### 4.1.3. Social participation.

Social engagement is a protective factor for cognitive function in individuals with CKD. The 2019 World Health Organization Guidelines for Reducing the Risk of Cognitive Impairment and Dementia indicate that low social engagement is a risk factor for cognitive decline or dementia [22]. Studies have shown that prolonged social isolation affects brain structure and cognitive function in older adults, while active participation in social activities helps improve cognitive function and may slow cognitive decline [23]. Caregivers should encourage CKD patients to participate in social activities according to their interests to reduce the risk of cognitive decline.

#### 4.1.4. Hemoglobin concentration.

In this study, lower hemoglobin concentrations were associated with an increased risk of CI in CKD patients. Research indicates that each 1 g/L increase in hemoglobin reduces the risk of mild cognitive impairment by 1.6% [20]. Low hemoglobin levels may impair cognitive function by inducing cerebral hypoxia or altering the microstructure of cerebral white matter [24,25]. Clinically, anemia is a common comorbidity in CKD patients, predisposing them to CI due to reduced oxygen-carrying capacity and inadequate cerebral oxygenation [26]. Regular monitoring of hemoglobin levels, iron supplementation, and erythropoietin therapy are essential to maintain normal hemoglobin levels. Preventing and managing vascular diseases such as atherosclerosis and thrombosis is critical to ensuring sufficient cerebral oxygenation by promoting vascular health. Proactive management of underlying conditions such as hypertension and diabetes is necessary to prevent further renal impairment and anemia, thereby mitigating its detrimental effects on cognitive function and improving quality of life in CKD patients.

### 4.2. Analysis of CI risk prediction model for CKD patients

Traditional CI risk prediction models are mainly for maintenance hemodialysis patients and are logistic regression models. The logistic regression algorithm has limitations. It cannot accurately identify relevant influencing factors and cannot fully utilize data characteristics, so the model accuracy is limited. The risk prediction models established based on machine learning algorithms in this study were explicitly designed for the field of CKD, providing an accurate and effective tool for CI risk prediction in this population, which can help medical workers to identify patients with high CI risk at an early stage, formulate an individualized intervention plan to slow down disease progression and improve patients’ prognosis, as well as provide a scientific basis for the development of cognitive health management and individualized intervention plans. The four risk prediction models in this study performed well overall, with AUC values ≥0.760, all with good differentiation and calibration, and the NNET model was able to predict the risk of CI occurrence in CKD patients better than the other three models. The SHAP method was used to visualize how the model worked so that clinical staff could make a general judgment about the risk of CI occurrence in CKD patients. The Streamlit library was utilized to deploy the model on a web page to achieve online real-time prediction of the model and to enhance the model’s utility.

A performance comparison of the machine learning model used in this study with the LR model from previous CKD research revealed the following: when compared to the LR-based Nomogram model from Literature 1 [27] (“Construction and Validation of a Predictive Model for the Onset of Cognitive Impairment Following Hemodialysis in Patients with Chronic Renal Failure.”), the AUC of the validation group for the LR model was 0.895, whereas the machine learning model (NNET model) in this study exhibited a higher AUC of 0.918. Similarly, when compared to the LR model from Literature 2 [28] (“Construction and Validation of a Risk Prediction Model for Mild Cognitive Impairment in Non-Dialysis Chronic Kidney Disease Patient”), the validation group AUC of 0.897 was lower than the 0.918 AUC achieved by the NNET model in this study.

### 4.3. Application of research

The successful deployment of this model through a web-based interface enhances its potential for widespread application in real-world clinical settings. The required input data are easily accessible, ensuring the model’s practicality. Specifically, age and education level can be directly retrieved from medical records and are readily available to physicians during routine consultations. Hemoglobin levels are included in regular blood monitoring and explicitly reported in laboratory results. Social participation is assessed through specific questions in the CHARLS questionnaire, which asks, “Have you engaged in any of the following activities in the past month?”—covering 11 social activities such as interacting with friends, playing mahjong, and card games. Physicians can simply review questionnaire responses, eliminating the need for additional testing equipment or specialized training. This tool enables healthcare professionals to conduct real-time risk assessments, facilitating timely interventions and ultimately improving patient outcomes.

### 4.4. Limitations

There are several limitations to this study. First, the relatively small sample size of this study may not sufficiently encompass all subtypes and stages of the disease progression in CKD patients, nor account for the intricate individual differences. This limitation could introduce bias in the feature patterns learned by the model, potentially impacting its applicability and generalizability to similar patient populations in different regions or healthcare settings. Second, because blood test indicators are not yet available for CHARLS data after 2015, this study used a cross-sectional design, and the causal relationships between variables and CIs may be questioned. Third, the four models have not undergone external validation due to constraints in research time and resources, representing a limitation of this study. In the future, external data will be collected, conditions permitting, to further assess the applicability and robustness of the models.

## 5. Conclusions

In conclusion, the four machine learning-based risk prediction models developed in this study serve as valuable tools for evaluating CI risk in CKD patients, with the NNET model demonstrating the best prediction efficacy. The SHAP package and Streamlit library enabled visual interpretation and web-based deployment of the models, facilitating real-time prediction and enhancing their practical applicability. Age, education level, social participation, and hemoglobin concentration were critical

factors influencing CI occurrence in CKD patients. Based on the output of the optimal model’s SHAP analysis, the most significant predictors are age, education level, and hemoglobin concentration. These help healthcare professionals to objectively assess the risk probability of CI in CKD patients and provide a basis for early intervention.
